# The Question of Population

**Published:** 1918-04-27

**Authors:** 


### THE QUESTION OF POPULATION.

### By a Member of the A.M.S.

The question of population is in the minds of men. This is evident to anyone who reads a newspaper. There is uneasiness about the matter. It was so before the war began, more in some lands than in others; it will be prominent after the war is over. Before the war came to complicate the matter, the nations with Western European civilisations were noting with uneasiness that their populations were increasing less rapidly than formerly. In France the native population was at a standstill. In the British Isles the birth-rate was falling, and was sustained by the breeding of foreign races settled in the towns and Irish. The valuable and efficient English and Scottish stocks had a more diminished birth-rate than gross figures displayed. The German Empire was warning its peoples that a fall of their birth-rate had begun, and was apprehensive because the fall was sudden and steep. The populace has not comprehended that the decline of the birth-rate is most marked in those elements of the populations that average most highly in physical and mental power combined, but discerning individuals have observed this portentous phenomenon.

There arose an outcry against the selfishness of the bachelors, the women were abused for failing in their duty to the race, committees were formed to try to keep alive as many as possible of the children born. "Save the precious babies, England's greatest wealth," and such were the catchwords. It is doubtful if many of the babies that can be saved alive only by the exertions of the charitable will prove a source of wealth to the nation or even repay the capital outlay.

Each nation has to face the question and answer it in its own way. Each nation must provide for such renewal of its population as shall keep it numerous enough to occupy and develop its territory, or other peoples will enter into possession. It may be necessary to make regulations that shall ensure that the average of physical and mental gifts of its individuals shall be maintained, or a nation may fall into decadence and come to consist of persons of lower average grade than their forefathers. Before answers are found, unpalatable truths may be arrayed and pleasing popular fallacies and assumptions may be swept away.

### The Popular Assumption.

Till recently no nation had reason to trouble about the matter of numbers. Men and women impelled by natural desire mated and families resulted. The death-rate was high but families were large, so the population grew greatly and no one was dismayed at the loss. "The Lord gave and the Lord taketh away" was the popular attitude. The assumption was that children are a blessing to all so fortunate as to possess them, and the more the children the more plenteous the blessing. No doubt certain owners of a quiverful privately questioned the truth of the assumption, but on the whole the assumption was received to be truth, and congratulations were showered on parents on the arrival of the last of a dozen as for the first-born son. Whatever the private views of the parents they accepted the inevitable and shouldered the burden of bringing up. The nation had population at the expense of individuals, not of the State, increased and was satisfied.

Unfortunately, the foundation assumption of this state of affairs is not sound. Every couple does not desire children, and still more couples do not desire as many children as possible. Although when children arrive they are usually loved and often brought up with care, still, their coming is not always eagerly looked forward to. Most of them are not desired beforehand, though hospitably received and entertained when they come.

### Prudent and Imprudent Hosts.

In primitive societies the guest is received gladly and welcomed to a share of plain fare and little-costing shelter, but when a community "raises its standard of living" the individual feels a strain to keep up to the level; guests are less welcome because burdensome and troublesome. Hospitality is offered less freely, not because hosts are less hospitable, but because the burden of a few guests is as great as formerly the burden of many. Thus it has fallen to be with prospective parents. The demands of the little guests, or the demands made for them by the community in written and unwritten codes, became so heavy as to be burdensome. The more prudent prospective hosts waited to invite guests till they felt certain of ability suitably to provide for their comfort and welfare. The imprudent still sent out invitations without provision, so when their guests arrived they had to scramble along anyhow, many of them coming off badly for food, shelter, and equipment for life. The community accepted as too commonplace for remark the conduct of the prudent, but regarding the other, became vocal. The imprudent hosts were abused for neglecting their guests, and ordered to give them sustenance and succour in accordance with the standard raised by the community and followed by the prudent. The prudent had the wherewithal to entertain guests only because for years before the arrival of them they had been making ready the means. They were therefore able and willing to entertain a certain number. The imprudent had no provision, and in some cases they were not able to secure, from day to day, even by hard work, sufficient to meet the standard of entertainment demanded of them by the community. In some cases the imprudent were not willing to work so hard as was necessary to meet the communal demand. In other cases, though able to meet the demand, they preferred to consume the bulk of their produce on their own pleasure, and gave to their guests less than they

**THE QUESTION OF POPULATION**—*(continued).*

could have afforded. A last class, often, no doubt, repenting folly, but cheered by a jolly company, set their teeth, struggled on gamely, and by silent, unremitting exertions kept their guests happy and healthy.

The community did not interfere with this last sort of imprudent persons; as to the others, when it found that some could not and others would not entertain guests according to standard, it began by punishing; but as it was evident this course did not avail it took another line and compelled the prudent to hand a portion of their provision to the imprudent for the suitable entertainment of their guests. The prudent considered hospitality a duty, so submitted, some willingly, some grudgingly, and of necessity delayed a little longer the issuing of invitations, sent out fewer, or took care the invitations should not be accepted.

The imprudent continued recklessly to issue invitations, and the more readily, perhaps, since a portion of the charges was shifted to others' shoulders; but still a portion was on them, and was grudged by many amongst them who had no real desire to entertain guests but wished only the pleasure of giving invitations. As more and more of these last learned the possibility of securing that invitation should not be followed by acceptance, the number of guests arriving at their houses lessened, as it already had lessened at the dwellings of the prudent. But guests were useful to the community, and now the community began to complain that as many guests as it required did not arrive, and to feel uneasy because of those that did come the most part came at the invitation of hosts whose invitations seldom were accepted by the best class of guests.

In parable, that is the situation. In fact, the community wants children, wants a certain standard of living kept up by its members, and wants the children reared and equipped for life according to standard. Many individuals are able and willing to bring up at their own charges a limited number of children, but some cannot and others will not support the burden of bringing up all the children they could beget or bear.

The State and Parenthood.

The number produced is no longer as many as the community desires. The State cannot any longer get all its population at the expense of individuals. It may become necessary that the community shall pay collectively for a number of children sufficient to make good the deficiency. If the community decides to take such a course, it rightly may come to decide what individuals are suitable to be the fathers and mothers of the children the cost of whom it is prepared to bear. Many vistas may be opened by action based on such a decision. The community may decide that no person, unless approved as a parent, shall have any part of the burden of bringing up his or her child borne by the State. The community may recognise child-bearing to be a national service, and pay selected women to do it. Regulations may come that shall lead to two systems, one matriarchal, the other patriarchal, existing side by side. However startling and contrary to present and former notions such ideas as these may be, they may have to be thought about if the community decides that it must have more children, and can have them only by paying for them, for it is not likely that the State will think it sound policy to pay persons not suited to be good parents to produce children not calculated to be capable citizens.

